# Structural investigation of C6/36 and Vero cell cultures infected with a Brazilian Zika virus

**DOI:** 10.1371/journal.pone.0184397

**Published:** 2017-09-12

**Authors:** Debora Ferreira Barreto-Vieira, Fernanda Cunha Jácome, Marcos Alexandre Nunes da Silva, Gabriela Cardoso Caldas, Ana Maria Bispo de Filippis, Patrícia Carvalho de Sequeira, Elen Mello de Souza, Audrien Alves Andrade, Pedro Paulo de Abreu Manso, Gisela Freitas Trindade, Sheila Maria Barbosa Lima, Ortrud Monika Barth

**Affiliations:** 1 Laboratory of Morphology and Viral Morphogenesis, Instituto Oswaldo Cruz, Fiocruz, Avenida Brasil, Rio de Janeiro, RJ, Brazil; 2 Laboratory of Flavivirus, Instituto Oswaldo Cruz, Fiocruz, Avenida Brasil, Rio de Janeiro, RJ, Brazil; 3 Laboratory of Pathology, Instituto Oswaldo Cruz, Fiocruz, Avenida Brasil, Rio de Janeiro, RJ, Brazil; 4 Laboratory of Virological Technology, Bio-Manguinhos, Avenida Brasil, Rio de Janeiro, RJ, Brazil; University of Minnesota College of Veterinary Medicine, UNITED STATES

## Abstract

Zika virus (ZIKV) is a member of the flavivirus genus, and its genome is approximately 10.8 kilobases of positive-strand RNA enclosed in a capsid and surrounded by a membrane. Studies on the replication dynamics of ZIKV are scarce, which limits the development of antiviral agents and vaccines directed against ZIKV. In this study, *Aedes albopictus* mosquito lineage cells (C6/36 cells) and African green monkey kidney epithelial cells (Vero cells) were inoculated with a ZIKV sample isolated from a Brazilian patient, and the infection was characterized by immunofluorescence staining, phase contrast light microscopy, transmission electron microscopy and real-time RT-PCR. The infection was observed in both cell lineages, and ZIKV particles were observed inside lysosomes, the rough endoplasmic reticulum and viroplasm-like structures. The susceptibility of C6/36 and Vero cells to ZIKV infection was demonstrated. Moreover, this study showed that part of the replicative cycle may occur within viroplasm-like structures, which has not been previously demonstrated in other flaviviruses.

## Introduction

The Zika virus (ZIKV) was first identified in the Zika forest, Uganda, in 1947 in samples taken from a captive, sentinel rhesus monkey [1] and was later detected in humans in Uganda and the United Republic of Tanzania in 1952 [1]. In 2007, ZIKV spread from Africa and Asia, and the first large outbreak of the disease occurred on the Island of Yap in the Federated States of Micronesia [2]. In 2013/2014, the virus caused outbreaks in French Polynesia, Easter Island, the Cook Islands, and New Caledonia [3]. The outbreak in French Polynesia generated thousands of suspected infections [4]. In early 2015, several cases of patients presenting such symptoms as mild fever, rash, conjunctivitis and arthralgia were reported in northeastern Brazil [5]. The introduction of ZIKV significantly impacted public health in the country. In 2015, an outbreak of ZIKV fever struck Brazil and other regions of the Americas, causing an estimated 1.3 million cases [5, 6, 7].

Although ZIKV infection has historically been characterized as a mild self-limiting febrile illness capable of causing a maculopapular rash [8], an abrupt rise in the number of babies born with microcephaly and other fetal malformations was reported in September of 2015 in the Northeastern Region of Brazil [9, 10], and this phenomenon was strongly associated with ZIKV infection. ZIKV was isolated from the amniotic fluid of a pregnant woman whose newborn had confirmed microcephaly [9, 11] and from the brain of a fetus with neurological abnormalities [7] to corroborate the hypothesis that neurological disorders are directly related to ZIKV infection. On February 1^st^ 2016, the World Health Organization declared that the recent association of ZIKV infection with outbreak of microcephaly and other neurological disorders constituted a Public Health Emergency of International Concern [4]. Recent studies have demonstrated significant death of neural stem cells infected with ZIKV, which provided direct evidence of ZIKV-mediated inhibition of fetal brain development [12].

ZIKV is a member of the flavivirus genus which also includes yellow fever virus (YFV), dengue virus (DENV), Japanese encephalitis virus (JEV), and West Nile virus (WNV) [13]. ZIKV is a positive-strand RNA virus enclosed in a capsid and surrounded by an envelope membrane. The use of cryoelectron microscopy to undertake a structural study of ZIKV particles showed that the overall viral architecture is similar to that of other flaviviruses [14]. This study also revealed that ZIKV surface proteins have tighter interactions than do DENV surface protein, thereby making ZIKV particles more stable than DENV particles. This structural stability of ZIKV may help it to survive in the harsh conditions of semen [15], saliva [16] and urine [17]. During flavivirus replication, virus binding and uptake are believed to involve receptor-mediated endocytosis via cellular receptors specific for viral envelope proteins. The low pH of the endosomal pathway induces fusion of the virion envelope with cellular membranes. Following uncoating of the nucleocapsid, the RNA genome is released into the cytoplasm. RNA replication occurs at the surface of the endoplasmic reticulum and virus assembles into the endoplasmic reticulum (ER) cisterns [18]. The virus particles are transferred to the Golgi apparatus where the prM protein is cleaved, and new virions are released by exocytosis [19]. One remarkable feature of flavivirus biology is the interaction between virus replication and cellular membranes. These viruses induce a marked proliferation and rearrangement of ER membranes to reconfigure them into a variety of vesicular structures that bulge into the ER lumen and serve as replication compartments. Research with Vero E6, Huh7, and 293T cells has demonstrated that ZIKV infection leads to major rearrangements of the infected cell cytoplasm, particularly remodeling of the ER [20, 21].

Identifying a ZIKV susceptible cell line that may enable viral isolation and identification, viral stock production, and testing of drug and vaccine candidates is of utmost importance [22]. It is also important to know the dynamics of viral replication, since the literature provides little data on the topic. In this study, aiming at verifying presence and replication cycle of ZIKV and ultrastructural cell alterations, C6/36 and Vero cells were experimentally infected with a ZIKV sample isolated from a Brazilian patient and morphologically analyzed by phase contrast light microscopy and transmission electron microscopy. Our analyses demonstrated that both cell lines were susceptible to ZIKV infection (see also Rossignol et al., 2017 [21] and Offerdahl et al., 2017 [23]); the presence of several viroplasm-like structures was observed in the perinuclear area. The results presented in our study highlight the importance for additional investigation of how ZIKV replicates, disseminates into tissues and transmits to other organisms to develop therapeutic approaches against this virus.

## Materials and methods

### Virus

The ZIKV sample used was isolated in C6/36 cells from Brazilian patient blood in 2015 by the Flavivirus Reference Laboratory, Instituto Oswaldo Cruz, Fiocruz (Human Research Ethic Committee protocol number: 59254116.0.1001.5262). C6/36 cell monolayers were inoculated with 100 μL of the patient sample and incubated for 1 hour (hr) at 28°C for adsorption. The monolayers were subsequently kept in Leibovitz medium (Cultilab) supplemented with 1% non-essential amino acids, 10% tryptose phosphate broth, and 2% fetal bovine serum (Cultilab). The cell cultures were maintained at 28°C. Cytopathic effects (CPE) were investigated by inverted light microscopy. The sample was tested by real-time RT-PCR using specific primers, and the complete genome sequence was deposited in GenBank under the accession number KX197205. The infected C6/36 cell culture supernatant was collected, and viral titration was performed in Vero cell culture by plaque assay. The viral titer was 2,8x10^8^ PFU/ml.

### Cells

#### C6/36 cells

Cell monolayers were inoculated with a multiplicity of infection (MOI) of 1 per ZIKV sample; the adsorption time was 1 hr at 28°C. The monolayers were subsequently maintained at 28°C in Leibovitz medium (Cultilab) supplemented with 1% non-essential amino acids, 10% tryptose phosphate broth, and 2% fetal bovine serum (Cultilab). CPEs were investigated at 24, 48 and 72 hr post-infection (p.i.) by inverted light microscopy.

#### Vero cells

Cell monolayers were inoculated with an MOI of 1 per ZIKV sample that was adsorbed onto the cells for 1 hr at 37°C. After the incubation period, Minimum Essential Eagle Medium (Cultilab), supplemented with 2% fetal bovine serum (Cultilab), 2 mM L-glutamine and 100 U/50 μg penicillin/streptomycin, was added, and the cells were cultured at 37°C in a 5% CO_2_ atmosphere. CPEs were investigated at 24, 48 and 72 hr p.i. by inverted light microscopy.

### Infection kinetics

#### Transmission electron microscopy and real-time RT-PCR analyses

Six 25 cm^2^-bottles were used for transmission electron microscopy and real-time RT-PCR analyses of each cell line. For each cell line, three bottles were inoculated with the ZIKV sample and fixed at 24, 48 and 72 hr p.i., and three bottles of uninfected cells, used as negative controls, were fixed at the same time as the infected cells (this experiment was performed in triplicate).

#### Indirect immunofluorescence assay

The cultures, seeded at a density of 2x10^5^ Vero cells/well and 5x10^5^ C6/36 cells/well on round glass coverslips immersed in 24-well culture plates, were inoculated with the ZIKV sample. The monolayers were fixed at 24, 48 and 72 hr p.i.

### Real-time RT-PCR

Viral RNA was extracted from the supernatant of Vero and C6/36 cell cultures collected 24, 48 and 72 hr p.i. using the QIAamp viral RNA minikit (Qiagen, Hilden, Germany). Quantitative RT-PCR was performed in duplicate with 10 μL of RNA in a final reaction volume of 50 μL using the GoTaq Probe one-step RT-qPCR system (PROMEGA) according to the manufacturer’s protocol. Primers and probes were described by Lanciotti et al., 2008 [24]. Each assay included a negative (nontemplate) control and standard curve in the range of 10e7 to 10 copies/reaction. The qPCR standard curve used consisted of a synthetic 273-base pair gene that encodes part of the NS5 and envelope ZIKV proteins inserted into a commercial plasmid (pUCIDT-KAN). Plasmid DNA was transformed into *E*. *coli* and purified followed by quantification using the Qubit system to estimate the number of copies according to the Avogadro formula.

### Indirect immunofluorescence assay

Cells grown on glass coverslips were fixed in 4% paraformaldehyde in 2X phosphate-buffered saline (PBS) solution for 20 min at 4°C, washed three times with PBS, permeabilized with 0.1% Triton X-100 diluted in PBS for 5 min, washed one more time and saturated with 3% bovine serum albumin for 30 min. Samples were incubated with mouse IgG2a monoclonal antibody against ZIKV envelope (E) protein (4G2) diluted in PBS (1:4) for 1 hr at room temperature. After washing twice with PBS, samples were incubated with the secondary antibody Alexa Fluor 488 (1:500) (Invitrogen) for 1 hr. After staining, samples were incubated with 10 μg/ml 4,6-diamidino-2-phenylindole (DAPI) (Sigma-Aldrich) for cell nucleus visualization to allow direct quantification of infection levels. Finally, the coverslips were fixed in 2.5% 1,4-diazabicyclo (2,2,2)-octane (DABCO) (Sigma-Aldrich) to prevent loss of fluorescence. Samples were examined immediately using a Zeiss photomicroscope equipped with epifluorescence (Zeiss Inc., Thornwood, NY). The number of infected host cells was counted in at least 200 host cells per duplicate. The percentage of infection was estimated by counting the number of infected host cells/number of total host cells*100. The results were expressed as the mean ± standard deviation of the infection percentage (%).

### Cell processing for transmission electron microscopy analysis

Cells were fixed in 1% glutaraldeyde in sodium cacodylate buffer (0.2 M, pH 7.2) (Electron Microscopy Science), post-fixed in 1% buffered osmium tetroxide (Electron Microscopy Science), dehydrated in acetone (Merck), embedded in epoxy resin (Electron Microscopy Science) and polymerized at 60°C over the course of three days [25, 26, 27, 28]. Ultrathin sections (50–70 nm thick) were obtained from the resin blocks. The sections were picked up using copper grids, stained with uranyl acetate (Electron Microscopy Science) and lead citrate (Electron Microscopy Science), and observed using a Jeol JEM 1011 transmission electron microscope.

## Results

### ZIKV RNA detection from C6/36 and Vero cell culture supernatants

To evaluate the ability of C6/36 and Vero cells to produce ZIKV progeny, real-time RT-PCR was performed on cell supernatant samples collected at different time-points of infection. As shown in Table 1, ZIKV RNA was detected in the supernatant samples from both inoculated cell lineages at all infection time points. Viral RNA detection in the C6/36 cell line was constant throughout all timepoints, with cycle threshold (C_t_) values ranging from 24.54 to 25.84 and a viral genome titer ranging from 5.80 to 5.90X10 copies/mL. Vero cells presented peak RNA viral titers at 72 hr p.i., with a C_t_ value of 22.58 and a viral genome titer of 5.74X10 copies/mL.

**Table 1:** Detection of ZIKV RNA and viral load quantification from C6/36 and Vero cell supernatants at 24, 48 and 72 hr p.i. by quantitative real-time RT-PCR.

	Vero Cells		C6/36 Cells	
Time (h.p.i.)	Ct	Log 10 copies/mL	Ct	Log 10 copies/mL
**24**	26.76	4.84	24.87	5.90
**48**	26.03	4.95	24.54	5.89
**72**	22.58	5.74	25.84	5.80

Cycle threshold (C_t_), hours post infection (h. p.i.).

### Presence of CPE and ZIKV envelope antigens in C6/36 and Vero cells

**Fig 1:**
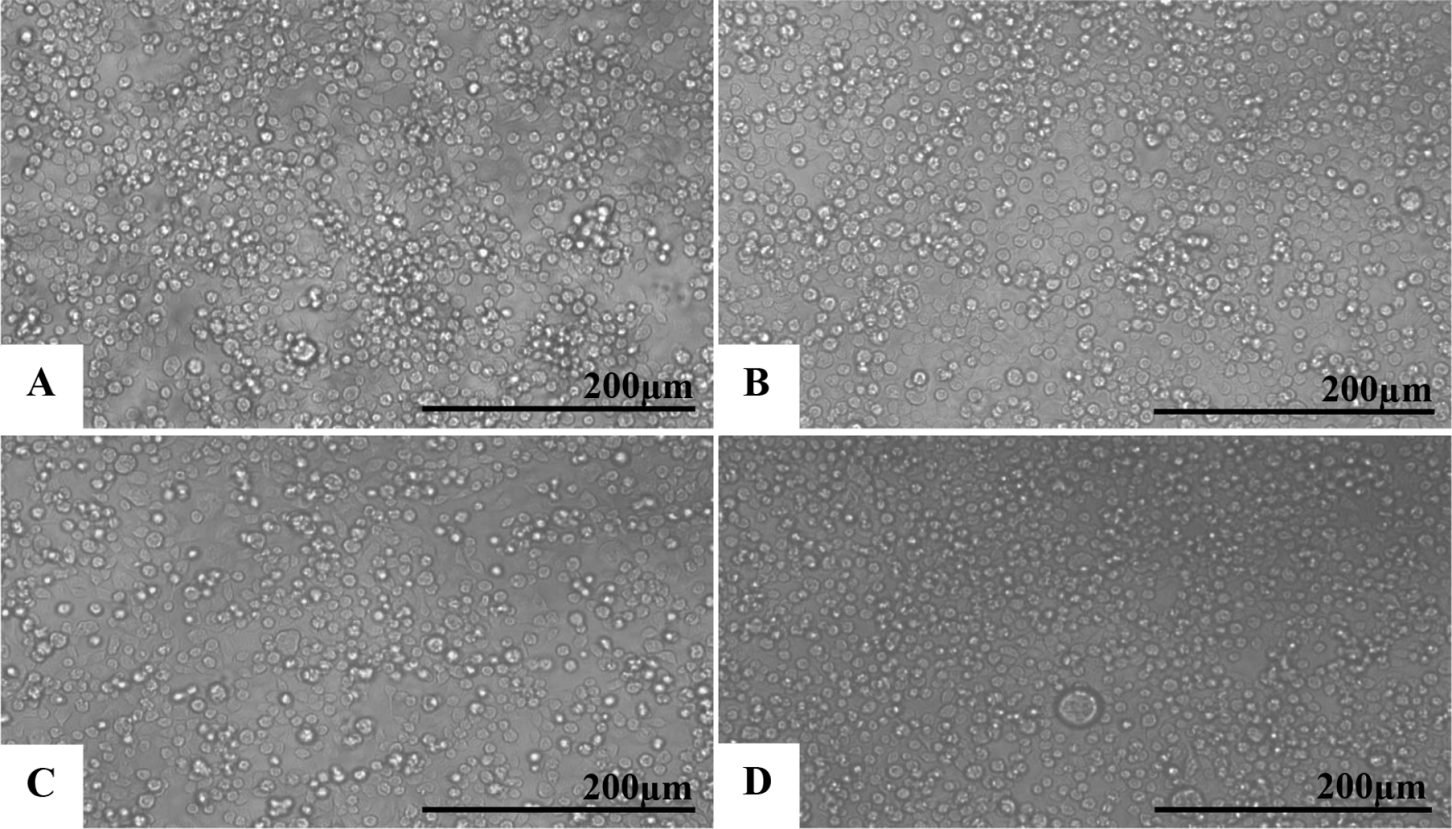
C6/36 mosquito cells infected with ZIKV analyzed by phase contrast light microscopy at different time points post infection. (A) Uninfected cells with no monolayer changes; (B) 24 hr p.i.; (C) 48 hr p.i.; (D) 72 hr p.i. Cell individualization, rounding and detachment as well as cytolysis were observed at all time points and were more evident at 72 hr p.i.

**Fig 2:**
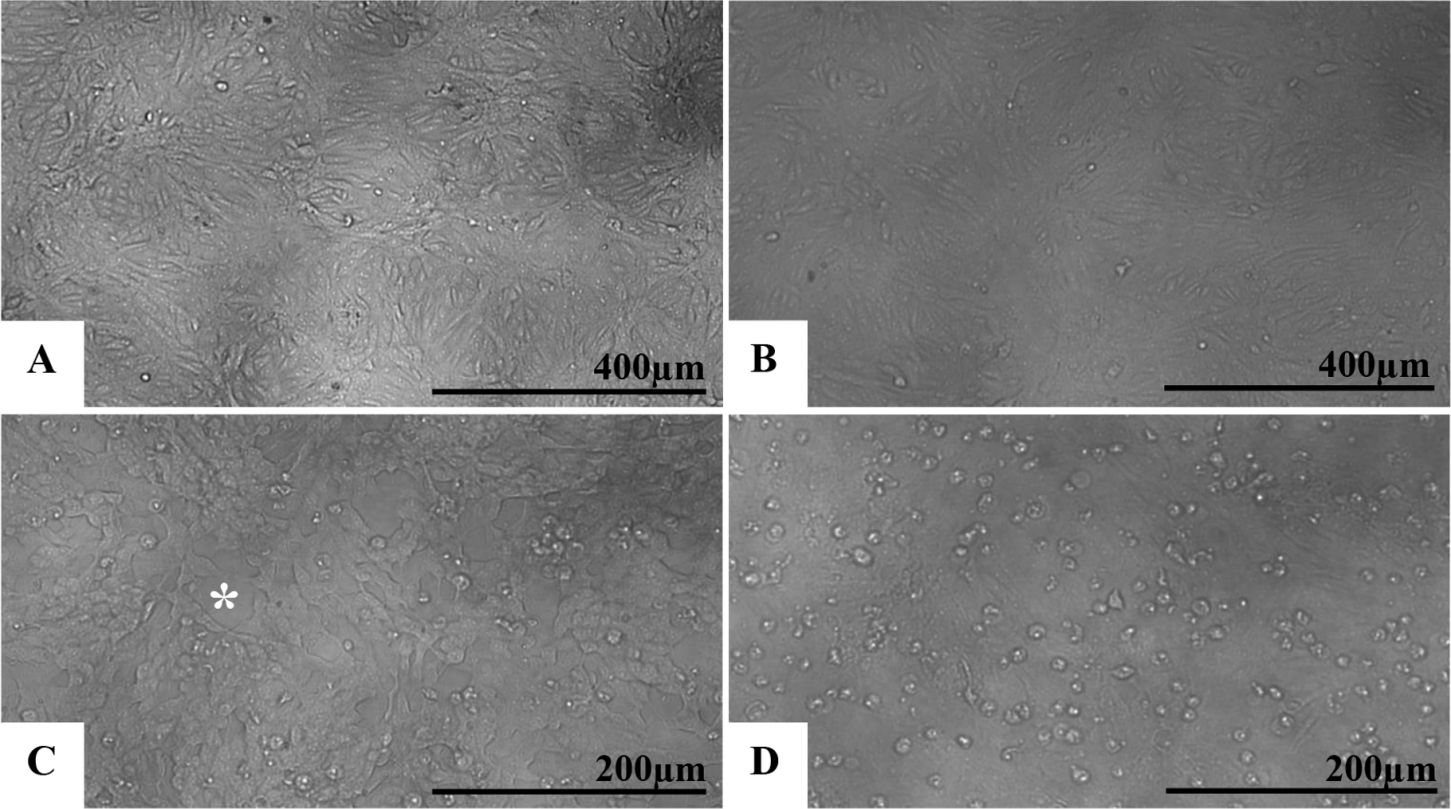
Vero cell monolayer infected with ZIKV analyzed by phase contrast light microscopy at different time points post infection. (A) Uninfected cells with no monolayer changes; (B) 24 hr p.i.; (C) 48 hr p.i.; (D) 72 hr p.i. Cell individualization, rounding and detachment as well as cytolysis were observed at all time points and were more evident at 72 hr p.i.

No changes were observed in the uninfected C6/36 and Vero cells (Figs 1A and 2A, respectively). In contrast, cells inoculated with the Brazilian patient ZIKV isolate showed cytolysis, cellular individualization, and a large number of detached cells in the supernatants sampled at 24 (Figs 1B and 2B), 48 (Figs 1C and 2C) and 72 hr p.i. (Figs 1D and 2D). These CPEs were more evident at 72 hr p. i. Syncytia were rarely observed and only present in the C6/36 cell cultures.

**Fig 3:**
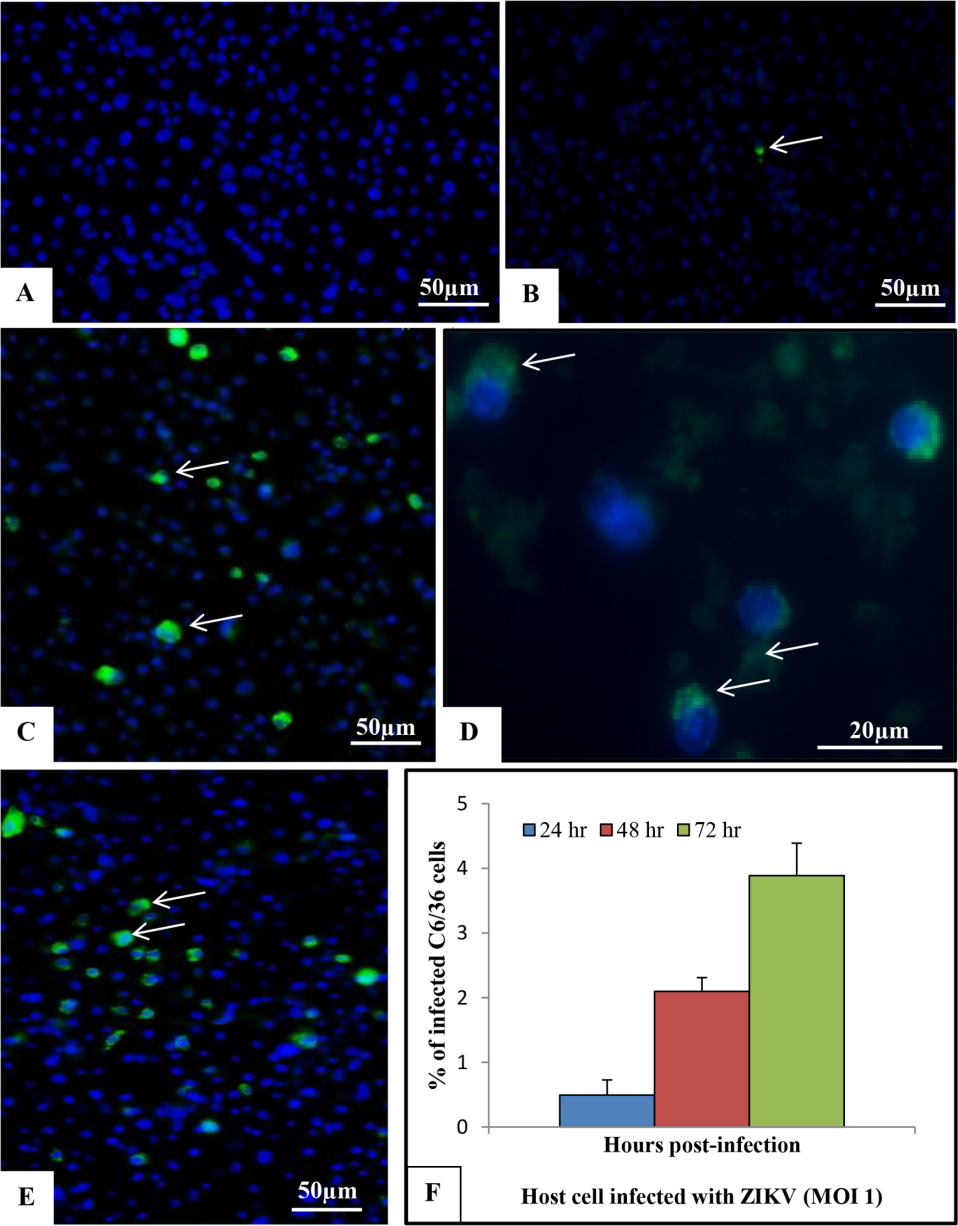
Immunolocalization of ZIKV E protein (4G2, green) [arrow] inside cytoplasm of C6/36 cells infected with ZIKV at different time points post infection. (A) Uninfected cells; (B) 24 hr p.i.;(C/D) 48 hr p. i.; (E) 72 hr p.i. The nuclei (N) were counterstained with DAPI (blue). (F) The graph represents the mean ± standard deviation of the infection percentage (%). The highest number of infected cells was observed at 72 hr p.i.

**Fig 4:**
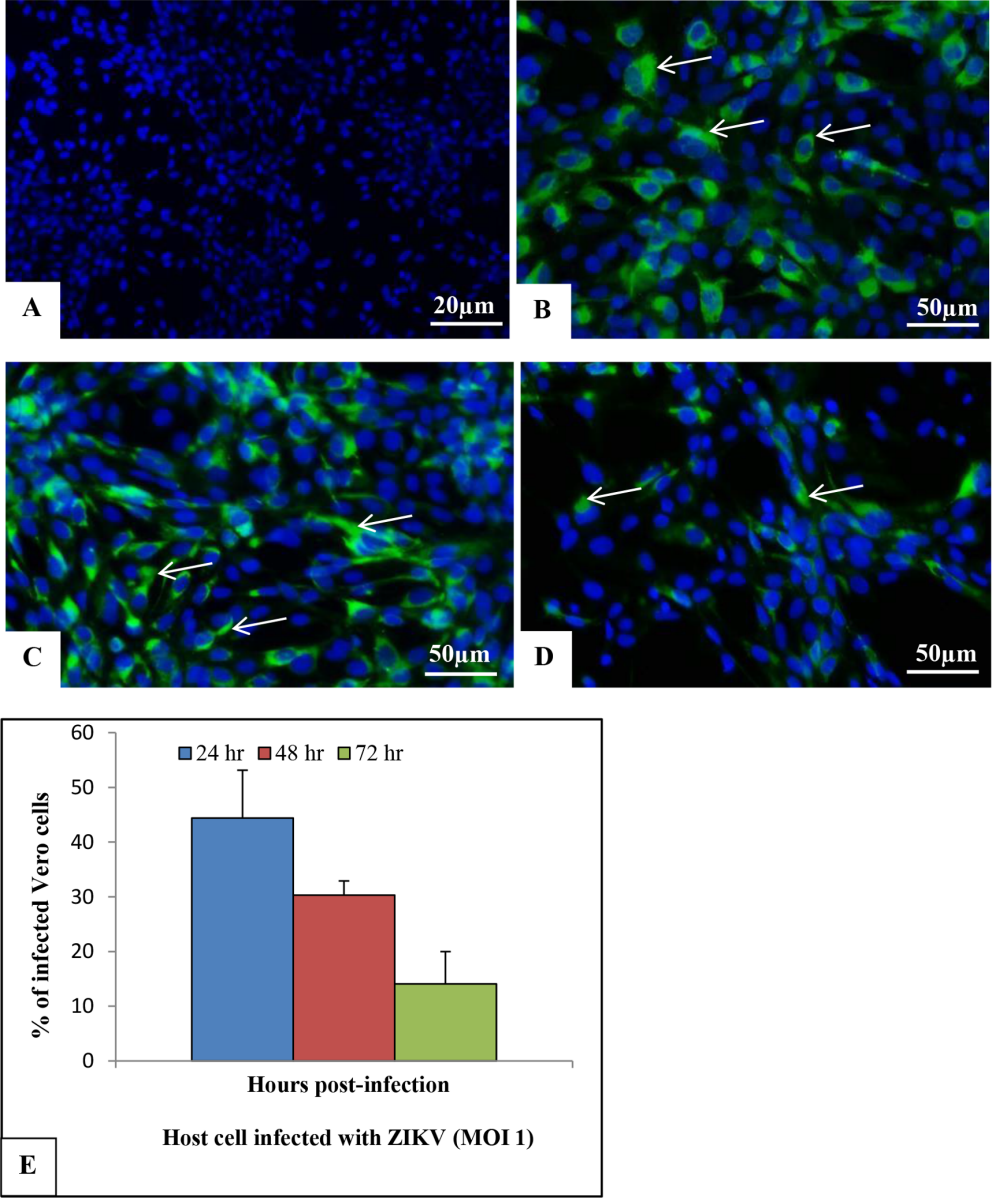
Immunolocalization of ZIKV E protein (4G2, green) [arrow] in the cytoplasm of Vero cells infected with ZIKV at different time points post-infection. (A) Uninfected cells; (B) 24 hr p.i; (C) 48 hr p.i.; (D) 72 hr p.i. The nuclei were counterstained with DAPI (blue). (E) The graph represents the mean ± standard deviation of the infection percentage (%). A higher number of infected cells was observed at 24 hr p.i.

The presence of viral envelope antigens was evaluated by indirect immunofluorescence assay. No staining was observed in the uninfected C6/36 and Vero cells (Figs 3A and 4A, respectively). In contrast, viral envelope protein expression was detected in C6/36 and Vero cell cytoplasm samples at 24 (Figs 3B and 4B), 48 (Figs 3C and 4C) and 72 hr p.i. (Figs 3D and 4D). The highest number of infected cells was observed at 72 hr p.i. in C6/36 cells (Fig 3E) and at 24 hr p.i. in Vero cells (Fig 4E).

### Ultrastructural analyses of C6/36 and Vero cells

**Fig 5:**
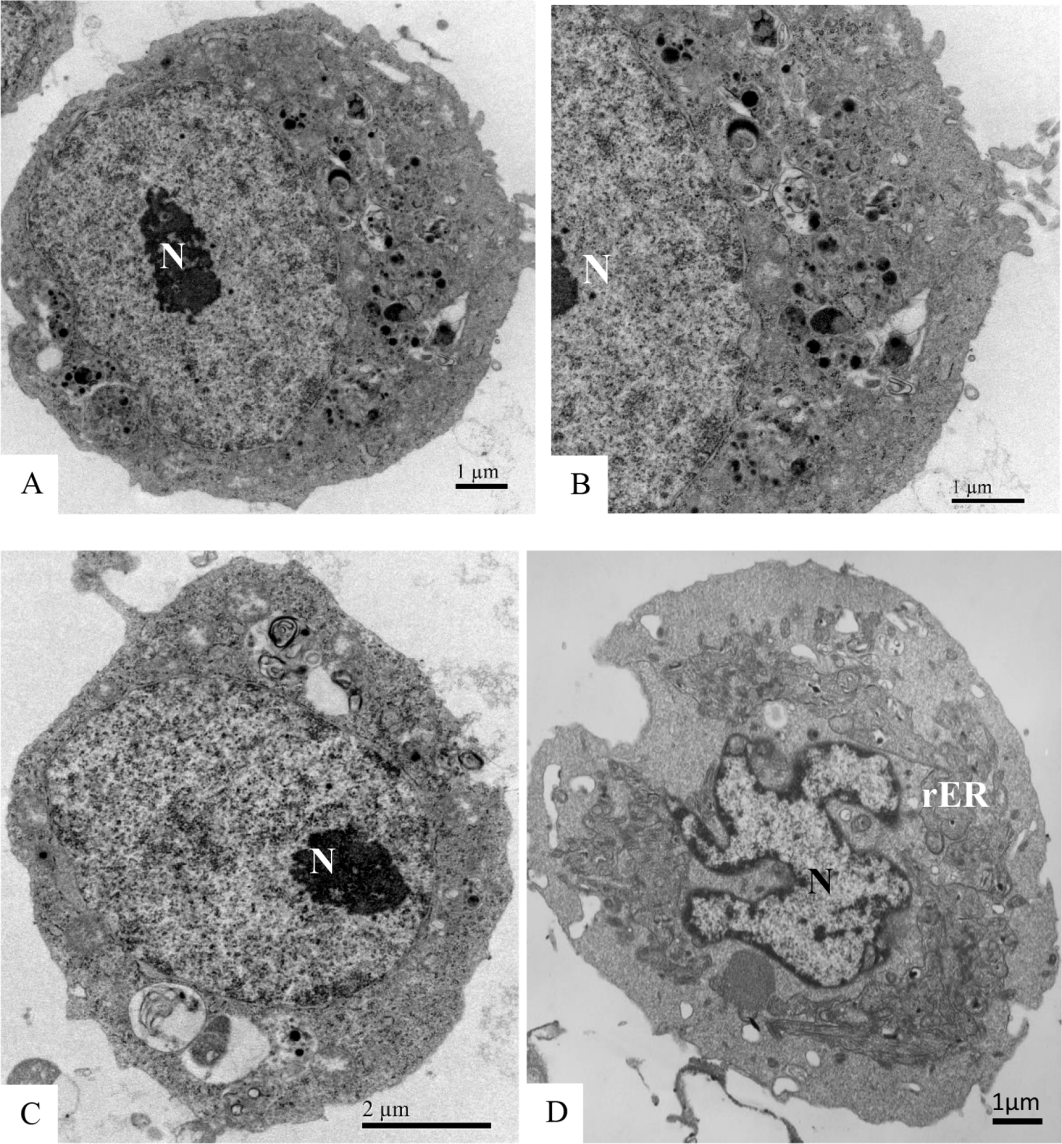
Uninfected C6/36 cells (negative controls) at different time points in culture. **No cellular ultrastructural alterations were observed.** (A–B) 24 hr of culture; (C) 48 hr of culture; (D) 72 hr of culture. Rough endoplasmic reticulum cisternae (rER), nucleus (N).

No ultrastructural changes were observed by electron transmission microscopy analysis of uninfected C6/36 cells (Fig 5A–5D).

**Fig 6:**
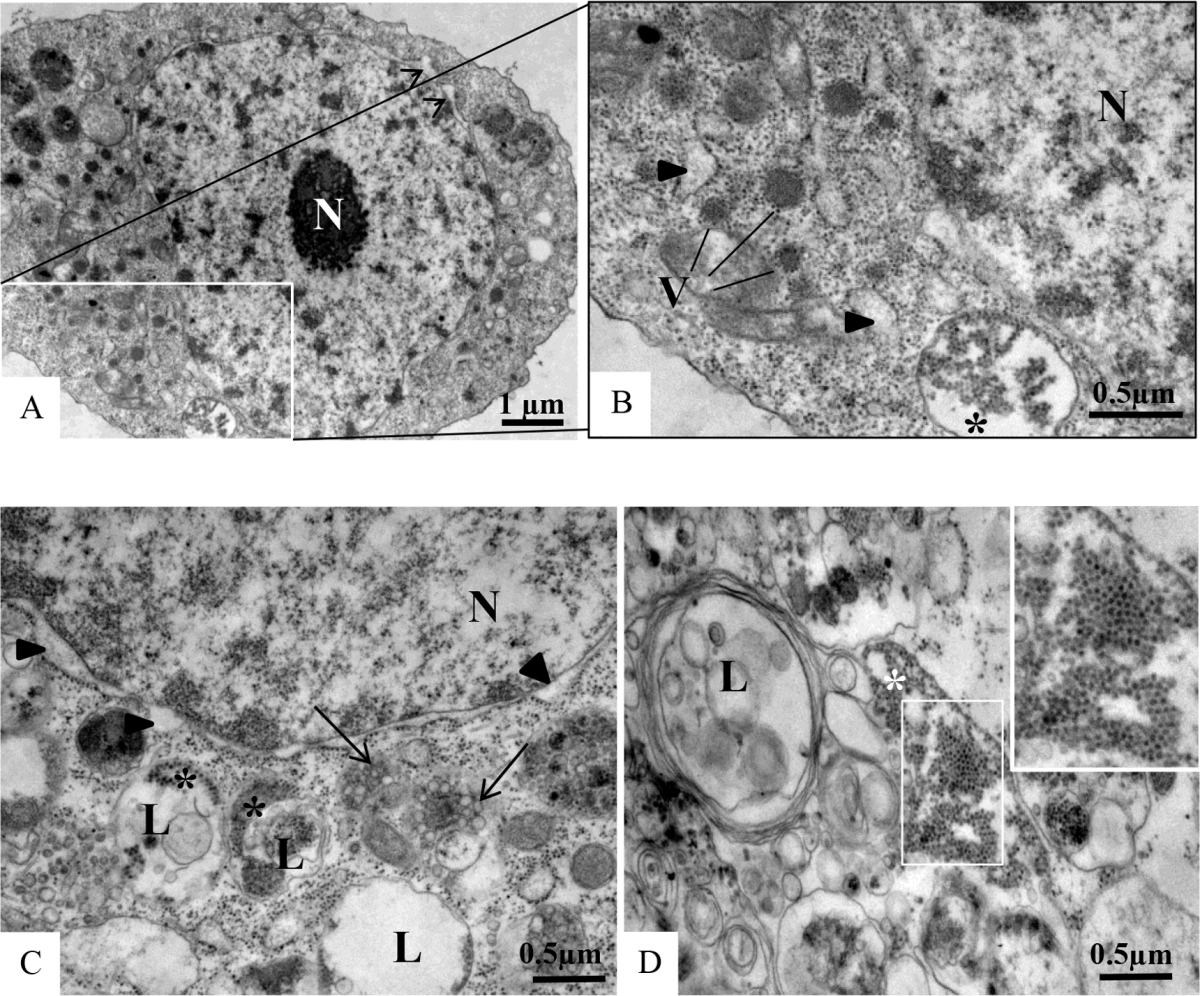
**C6/36 cells infected with ZIKV analyzed by transmission electron microscopy (TEM) at different time points post-infection (A-B: 48 hr p.i., C-D: 72 hr p.i.).** Several large viroplasm-like perinuclear compartments (V) (A-B) and ZIKV particles (*) measuring approximately 40–50 nm in diameter in the endoplasmic reticulum cisternae (RER) (A, B) and in lysosomes (L) (C-D) were observed. Nucleocapsids were observed inside the rER (D). Thickening of the nuclear membrane and rough endoplasmic reticulum cisternae (rER) (black head arrow) (C), numerous lysosomes (L) (C, D) and vesicular compartments associated with rER (arrow) (C) measuring approximately 100 nm in diameter were observed. Nucleus (N).

**Fig 7:**
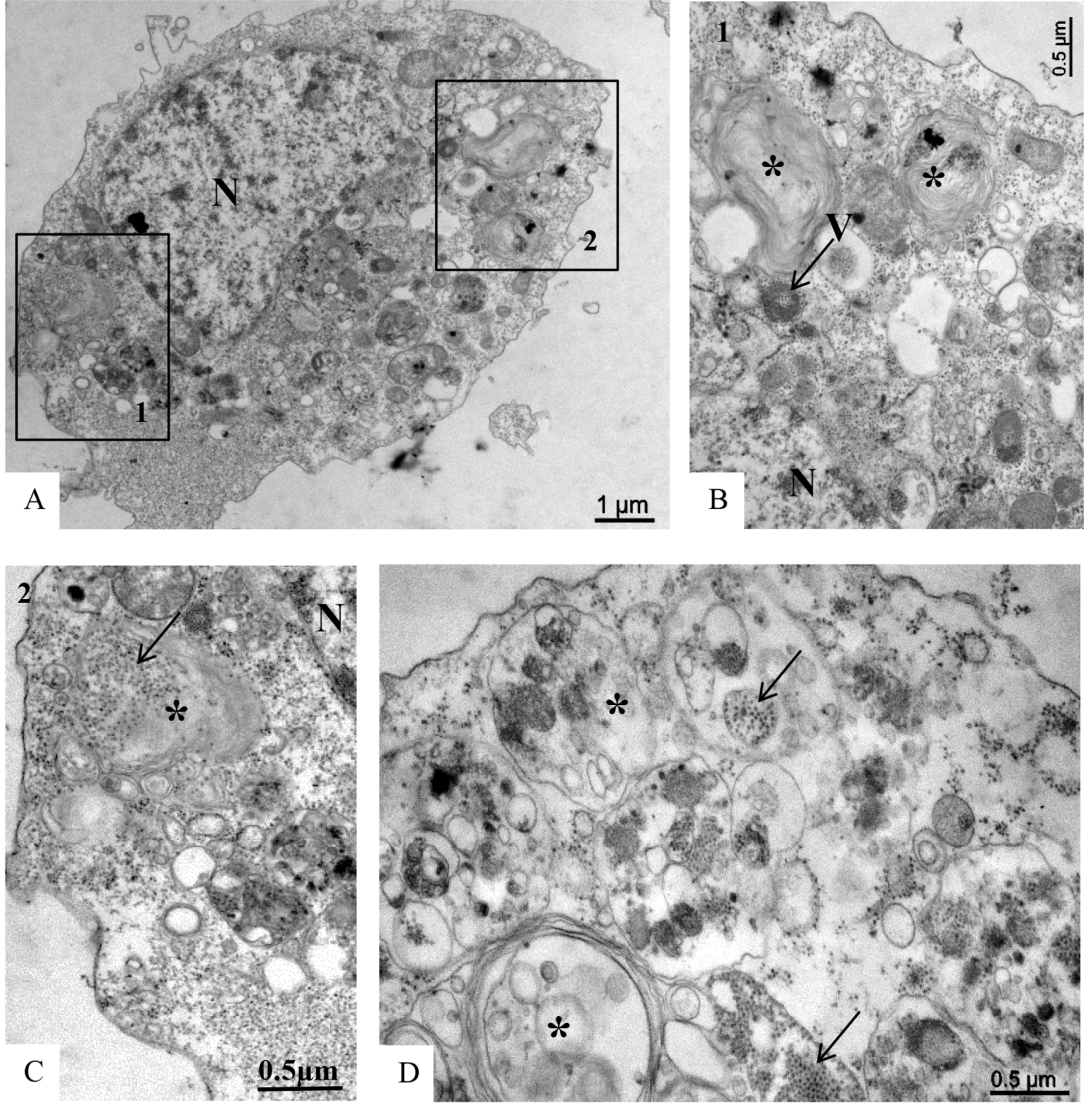
**C6/36 cells infected with ZIKV at different time points post-infection (A-C: 48 hr p.i., D: 72 hr p.i.).** The presence of several phagasomes (*) was observed. Marked areas of the image A (numbers 1 and 2). Virus particles (arrow) inside cytoplasmic vesicles. Nucleus (N), viroplasm-like perinuclear compartments (V).

**Fig 8:**
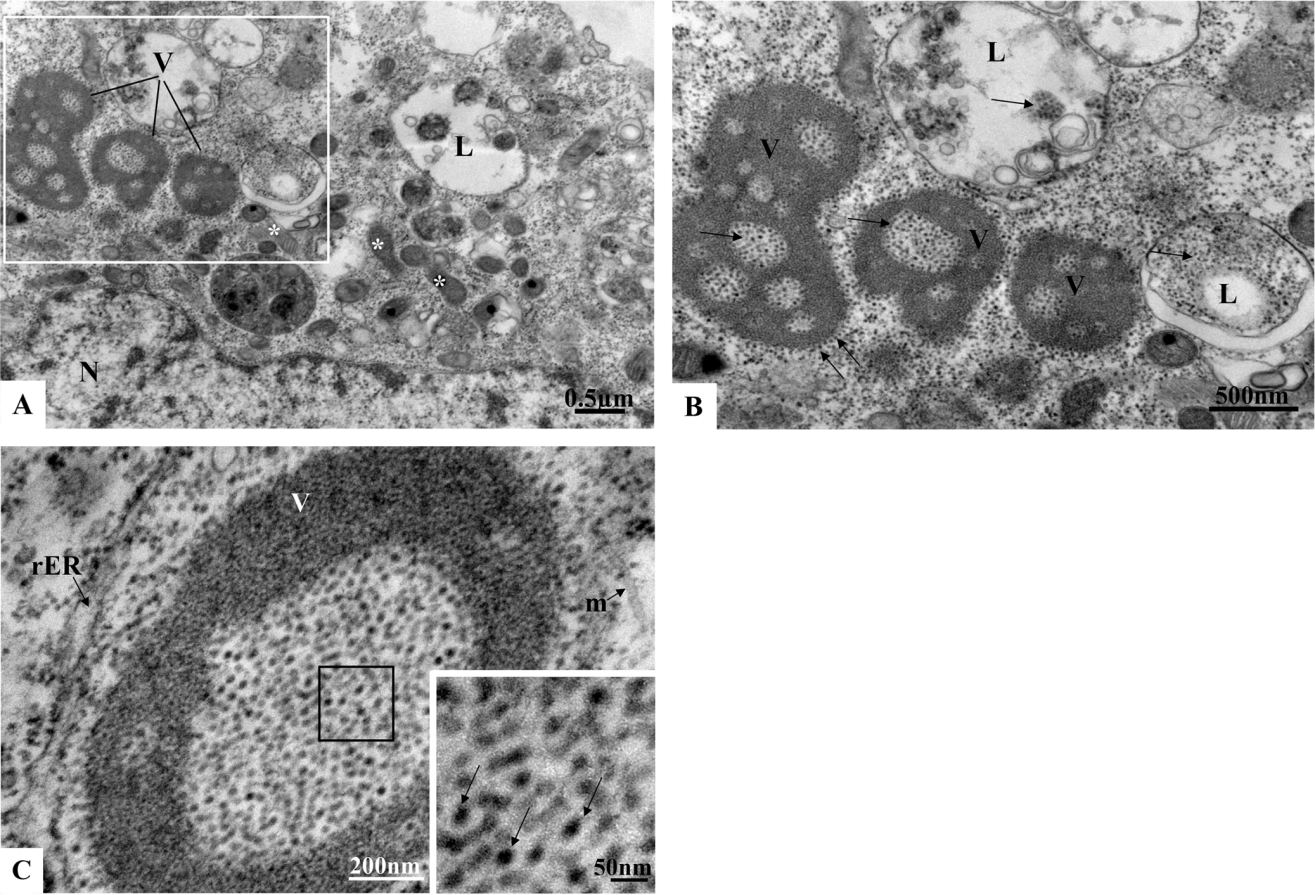
Increasing magnifications of ZIKV-infected C6/36 cells at 48 hr p.i. Several large viroplasm-like perinuclear compartments (V) containing ZIKV particles in the lumen (arrow) were observed (A, B, C); ZIKV particles were also observed in lysosomes (L). Rough endoplasmic reticulum cisternae (rER), mitochondria (*), microtubules (m). N = Nucleus.

C6/36 cells infected with ZIKV were examined for morphological alterations at all times of infection. The predominant changes associated with ZIKV infection were an increased number of thickened ribosomes (Fig 6A and 6B); numerous myelin figures, phagosomes (Fig 7A) and lysosomes (Fig 8B); thickening of the nuclear membrane and rough endoplasmic reticulum (Fig 6C); and development of vesicular compartments, measuring approximately 100 nm in diameter, associated with the rough endoplasmic reticulum (Fig 6C). Several large viroplasm-like compartments (Figs 6A, 6B, 7A and 8A–8C), localized to the perinuclear area together with periphery rough endoplasmic reticulum (Fig 8C), mitochondria (Fig 8A) and microtubules (Fig 8C), were also observed. ZIKV particles were observed inside lysosomes (Fig 6C and 6D) and rough endoplasmic reticulum (Fig 6A and 6B), as well as inside the the viroplasm-like structures (Fig 8A–8C). Viral nucleocapsids were only observed inside cisterns of the endoplasmic reticulum (Fig 6D).

**Fig 9:**
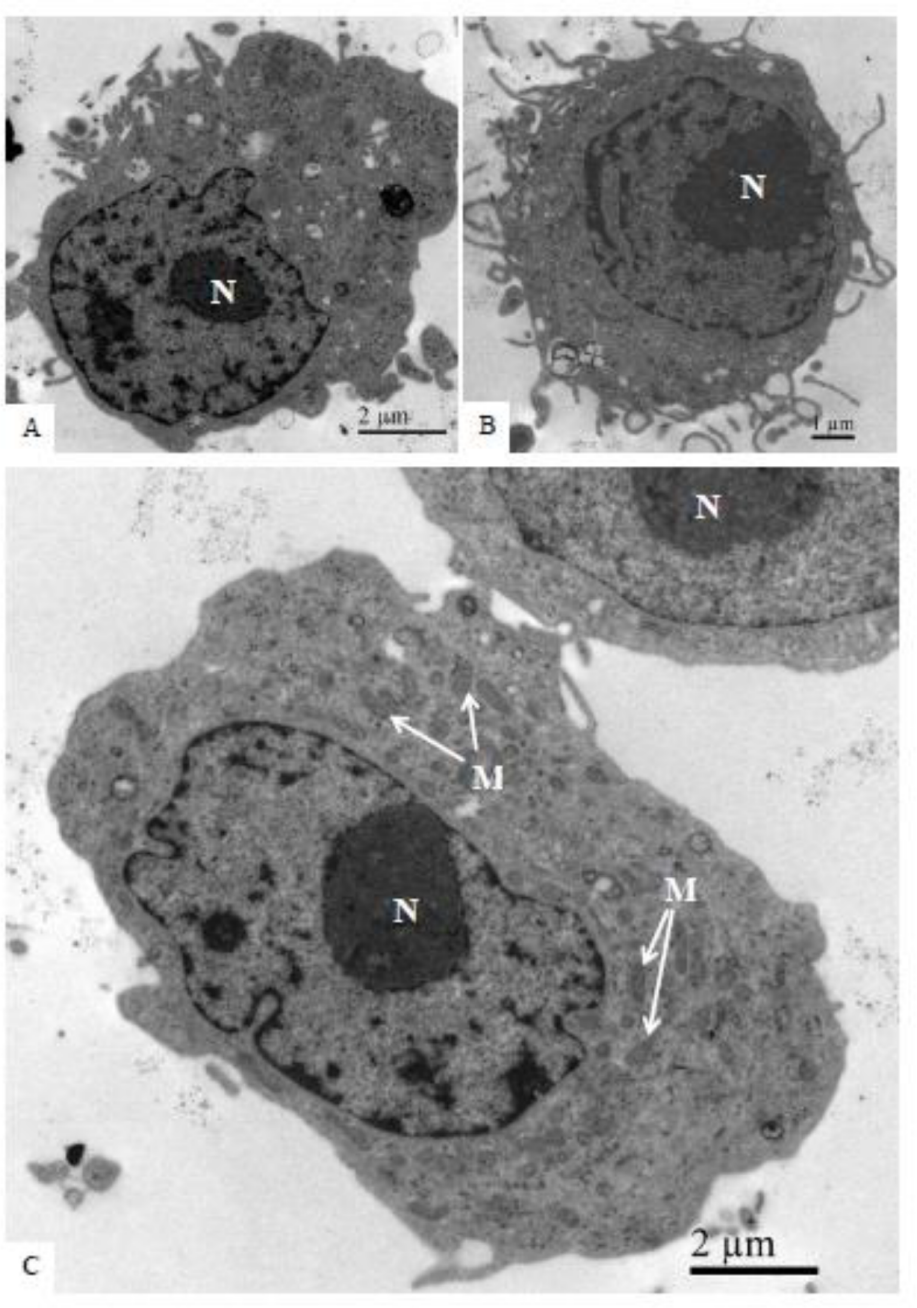
Uninfected Vero cells (negative controls) at different culture time points. No cellular ultrastructural alterations were observed. (A) 24 hr of culture; (B) 48 hr of cultures; (C): 72 hr of culture. Nucleus (N), mitochondria (M).

**Fig 10:**
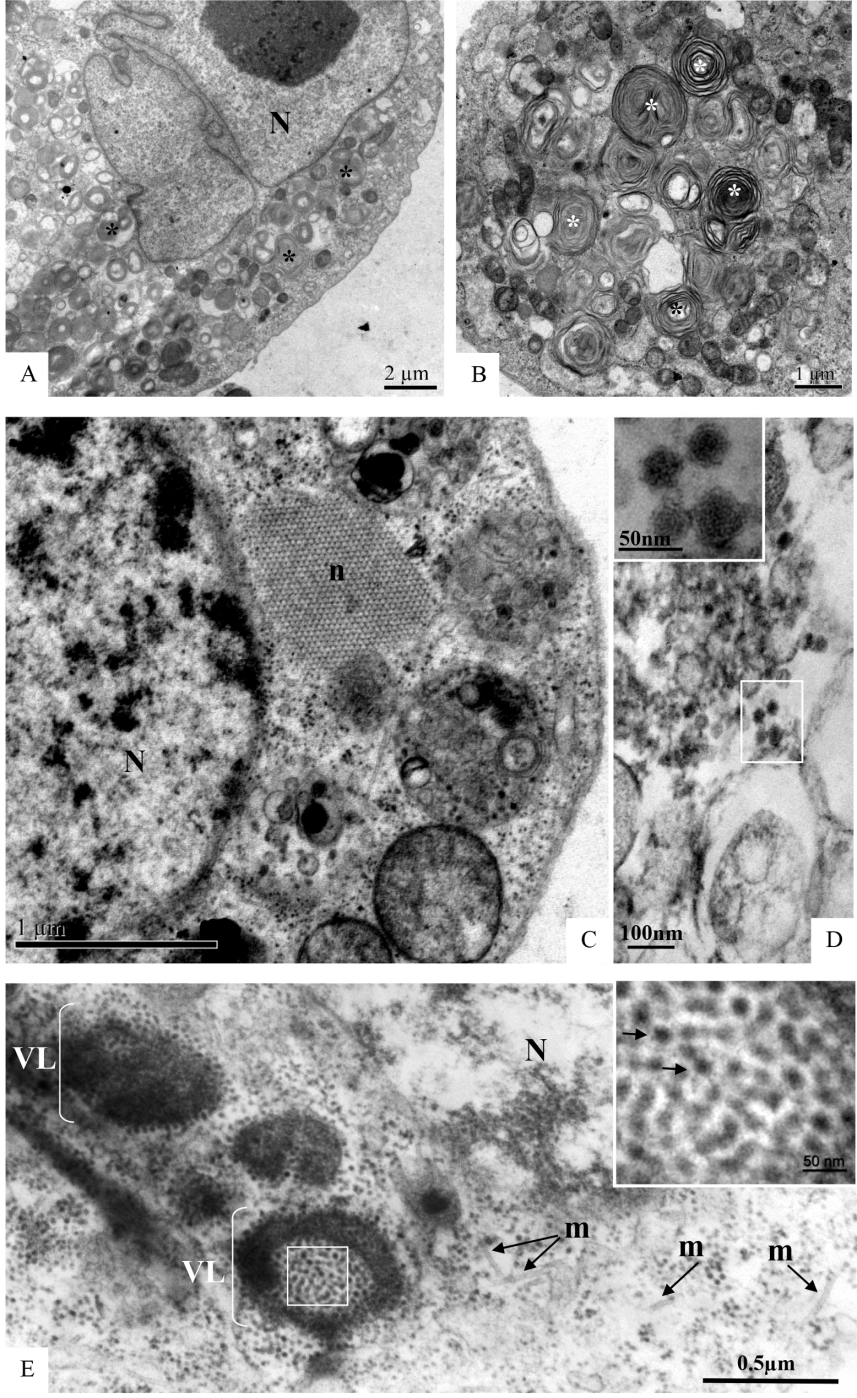
**Vero cell monolayers infected with ZIKV analyzed by transmission electron microscopy at 24 hr (A-D) and 72 hr p.i. (E)**. Numerous phagosomes (*)(A-B), nucleocapsids (n) inside the rough endoplasmic reticulum cisternae rER (C). ZIKV particles (black arrow) were observed inside in cytoplasm vesicles (D) and in lumen of electron dense viroplasm-like structures (VL) (E). Nucleus (N), microtubules (m).

No ultrastructural changes were observed of uninfected Vero cells (Fig 9A–9C). Morphological alterations observed in the Vero cell monolayers at all infection time points included increased numbers of ribosomes, phagosomes (Fig 10A and 10B), cytoplasm vacuolization, and numerous lysosomes containing ZIKV particles were similar to those observed in C6/36 cells. ZIKV particles were observed inside cytoplasmic vesicles (Fig 10D) and inside electron-dense viroplasm-like structures (Fig 10E). Viral nucleocapsids were observed inside the cisterns of the endoplasmic reticulum (Fig 10C).

## Discussion

ZIKV is an emerging arbovirus that causes an *Aedes* mosquito-borne disease [29]. The recent outbreak in Brazil has attracted international attention, not only because of the growing infected population but also due to the enhanced severity of clinical sequelae [30]. Zika fever has recently become a “Public Health Emergency of International Concern” due to the dramatic increase in cases of prenatal microcephaly and Guillain-Barré Syndrome in regions where ZIKV is endemic [31].

The standardization of an effective ZIKV *in vitro* experimental model is critical for studying the mechanisms of ZIKV replication as well as for developing effective drugs and vaccines. In this regard, and aiming to verify the presence of ZIKV and its replication, C6/36 and Vero cells were infected with a ZIKV sample isolated from a Brazilian patient and morphologically analyzed.

In this study, C6/36 and Vero cells were found to be highly permissive to ZIKV infection. The inoculation of these cells with a Brazilian ZIKV sample resulted in the rapid release of high number of RNA copies into the cell supernatant, indicating active viral replication. ZIKV-NS1 protein expression was observed by indirect immunofluorescence assay in both cell lineages. The highest number of infected cells was observed at 72 hr p.i. in C6/36 cells and at 24 hr p.i. in Vero cells. These data corroborate the study by Chan et al., 2016 [32], who observed susceptibility of differential cell lines to ZIKV infection. The first cell lines developed from *Ae*. *albopictus*, the C6/36 cell line (originally known as the ATC-15 cell line), were generated from larvae in the mid-1960s. Derivatives of the C6/36 cell line have been widely used to study the relationship between arboviruses and mosquito vectors [33]. We believe that the fact that C6/36 cells are better adapted to arboviruses has resulted in the infection profile demonstrated in this work. In these cells, the experimental data showed increasing infection over time. In contrast, Vero cells were more susceptible to ZIKV infection since high percentages of infection were observed at 24 hr p.i. and decreased thereafter, probably due to infection-induced cell death; our morphological analysis show empty spaces in the monolayers (Fig 4D) during the course of the experiment. The Vero cell susceptibility was verified by Nikolay et al., 2017 [34], in a study where cells were infected with different Brazilian ZIKV samples; the virus titers ranged from 1.2×10^6^ PFU/mL to 2.8×10^6^ PFU/mL.

Our analyses showed that ZIKV infection induced autophagy in C6/36 and Vero cells, as demonstrated by the presence of autophagosome vesicles. Autophagy usually has a protective role by removing damaged organelles and protein aggregates and maintaining cellular homeostasis [35]. Different from the degradative autophagy (fusion with the lysosome), the secretory autophagy pathway may cause viral particle expulsion or secretion [36]. The autophagy process can be subverted by viruses [29]. This is true for several arboviruses, including DENV [37, 38], Chikungunya virus [39], and JEV [40]. In poliovirus, the autophagosome formation can increase viral load, suggesting that autophagic machinery plays a favorable role in viral replication [41]. These viruses use components of the autophagy pathway to promote their replication and dissemination by clearing cells through multiple mechanisms. In this regard, autophagy may thus have both pro- and antiviral effects. Autophagosomes were observed in human skin fibroblast cells infected with ZIKV. It was also observed that the specific autophagy inhibitor 3-methyladenine strongly reduced ZIKV copy numbers in the infected fibroblast cells [29].

Spherical ZIKV, which is approximately 50 nm in diameter, were detected by transmission electron microscopy in C6/36 and Vero cell lineages infected with Brazilian samples inside lysosomes, rough endoplasmic reticulum and viroplasm-like structures. Nucleocapsids were observed inside ER cisterns. Previous studies demonstrated that besides C6/36 cells, skin immune cells, including dermal fibroblasts, epidermal keratinocytes, and immature dendritic cells, were also permissive to ZIKV infection [29, 42, 23]. Vesicular compartments measuring approximately 100 nm in diameter and associated with the ER were also observed in the present study. Electron microscopy analysis of ZIKV-infected primary skin fibroblasts showed the presence of membrane vesicles 70 to 100 nm in size that were located in intimate association with the ER, indicating that ZIKV replication occurs in close association with host cell membranes [29]. Such vesicular compartments were commonly observed in C6/36 cells infected with DENV [43, 44, 45]. YFV, WNV and DENV generate ER invaginations measuring 80–100 nm in diameter. Tomographic reconstructions of the membranes exposed to DENV show a continuous ER membrane network connected to spherical vesicles and convoluted membranes [46]. Virus-induced vesicles contain replicase proteins and dsRNA and are found within the ER lumen. Most vesicles have double membranes, suggesting that they are formed from invaginations into ER cisternae [46, 47].

For the first time, viroplasm-like structures were observed in cytoplasm of C6/36 and Vero cells infected with a Brazilian ZIKV strain through morphological analysis by transmission electron microscopy. ZIKV particles measuring approximately 50 nm were observed in the lumen of these structures. Many viruses replicate within subcellular microenvironments or ‘mini-organelles’ known as virus factories or ‘viroplasms’. Formation of these structures involves rearrangement of host cell membranes and cytoskeleton and induces a ‘cytopathic effect’ indicative of virus infection. It is generally believed that factories and viroplasms create a platform to concentrate replicase proteins, virus genomes and host proteins required for replication and at the same time physically separate replication sites from a myriad of cellular antiviral defenses [47]. Such structures have been reported in many unrelated groups of eukaryotic viruses whose replication occurs within cellular cytoplasm. Viroplasms have been observed in cauliflower mosaic virus [48], rotavirus [49, 50], vaccinia virus [51], and the rice dwarf virus [52]. To date, viroplasms have not been associated with flavivirus replication cycle. Viral replication, protein synthesis and assembly require a considerable amount of energy, which is provided by large clusters of mitochondria at the viroplasm periphery. The viroplasms observed in the present study were localized to the perinuclear area and contained abundant rough endoplasmic reticulum, mitochondria and microtubules in near proximity. A virus factory is often enclosed by a membrane derived from the rough endoplasmic reticulum or by cytoskeletal elements [53, 54]. Cortese et al., 2017 [20], showed that ZIKV infection in both human hepatoma and neuronal progenitor cells induced drastic structural modifications of the cellular architecture.

The susceptibility of C6/36 and Vero cells to infection with a ZIKV sample isolated from a Brazilian patient was confirmed in the present study. In addition, the dynamics of ZIKV replication were investigated and presented as different from what is commonly observed during replication by other flaviviruses, since part of the replication cycle of this virus can occur in viroplasm-like structures.

Further studies are necessary to investigate the relationship between viroplasm-like structures and ZIKV replication dynamics. The data presented in this study are important for use in the development of model systems to evaluate therapeutic approaches.
